# Effects of Virtual Reality-Based Distraction of Pain, Fear, and Anxiety During Needle-Related Procedures in Children and Adolescents

**DOI:** 10.3389/fpsyg.2022.842847

**Published:** 2022-04-19

**Authors:** Yan Wang, Liangmei Guo, Xinjuan Xiong

**Affiliations:** ^1^Emergency Department, General Hospital of Xinjiang Military Region of People's Liberation Army, Ürümqi, China; ^2^Neurology Department, The Second Medical Center & National Clinical Research Center for Geriatric Diseases, Chinese People's Liberation Army (PLA) General Hospital, Beijing, China; ^3^Department of Burns and Plastic Surgery, The 960th Hospital of the Chinese People's Liberation Army, Jinan, China

**Keywords:** virtual reality, needles, pain, fear, anxiety

## Abstract

**Background:**

Needle-related pain, fear, and anxiety can be a deterrent to treatments in children and adolescents. Virtual reality (VR) can be used to manage the poor experience of needle procedures.

**Objective:**

This meta-analysis aimed to examine the effects of VR on pain, fear, and anxiety related to needle procedures in children and adolescents.

**Methods:**

PubMed, EMBASE, and the Cochrane Library were searched for potentially eligible studies published up to June 2021. The outcomes were pain assessed by the Wong-Baker Faces Pain Scale (WBS) or Faces Pain Scale-Revised (FPS-R), and Visual Analog Scale (VAS), fear assessed by Children's Fear Scale (CFS), and anxiety assessed by Children's Anxiety Meter (CAM), VAS, or CFS. Because of expected heterogeneity among studies, all analyses were conducted using the random-effects model.

**Results:**

Ten studies were included (571 children in the VR group and 575 in the control group). Based on the WBS, VR reduced pain, either self-reported (WMD = −2.17, 95%CI: −3.37, −0.97), parent-reported (WMD = −3.52, 95%CI: −4.62, −2.42), nurse-reported (WMD = −3.29, 95%CI: −5.59, −0.99), and physician/investigator-reported (WMD = −3.48, 95%CI: −5.93, −1.04). Using the FPS-R, VR reduced needle-related pain compared with controls (WMD = −0.85, 95%CI: −1.64, −0.06). Similar results were observed for fear (children/adolescents: WMD = −1.52, 95%CI: −2.18, −0.86; parents: WMD = −1.71, 95%CI: −2.30, −1.13; nurses: WMD = −1.55, 95%CI: −2.47, −0.63; physicians/investigators: WMD = −0.59, 95%CI: −1.00, −0.18) and anxiety (self-reported: WMD = −2.79, 95%CI: −4.07, −1.54; parent-reported: WMD = −3.87, 95%CI: −5.58, −2.15; nurse-reported: WMD = −4.64, 95%CI: −6.56, −2.71; physician/investigator-reported: WMD = −2.06, 95%CI: −4.13, −0.00).

**Conclusion:**

A VR-based intervention could reduce needle-related pain, fear, and anxiety in children and adolescents.

## Introduction

Needles are involved in several medical procedures, but needles induce fear in many individuals, notably children (Orenius et al., 2018). Still, punctures are routinely required (often repeatedly) for health checks, vaccines, and the monitoring and treatment of many medical conditions. Balancing the treatment of various conditions, life-threatening or not, with the fear and anxiety imposed by the treatments is an important challenge for the healthcare community. Needle fear can progress to blood phobia and needle phobia, particularly in children, leading to treatment avoidance and poor adherence, contributing to a risk of poor medical outcomes (Orenius et al., 2018).

Distraction is a common non-pharmacological method for managing pain perception during puncture (Eijlers et al., 2019). Distraction tools such as kaleidoscopes, distraction cards, buzzers, listening to music, and video games effectively manage pain perception (Dahlquist et al., 2009; Hartling et al., 2013; Gerceker et al., 2018; Inan and Inal, 2019; Koc Ozkan and Polat, 2020). Recently, many studies showed that virtual reality (VR) could be used as a distraction method to successfully reduce procedural pain in children (Piskorz and Czub, 2018; Chan et al., 2019; Ozalp Gerceker et al., 2020). VR can be used to manage several painful experiences in children, including punctures, changing dressings, cleaning and draping burns, and management of chronic and acute pain (Sharar et al., 2008; Atzori et al., 2018; Birnie et al., 2018; Chan et al., 2019; Toledo Del Castillo et al., 2019; Ozalp Gerceker et al., 2020). VR involves the active participation of the patients in a task requiring cognitive or behavioral functioning, thereby distracting the brain from pain (Dumoulin et al., 2019). Indeed, a successful distraction device provides multisensory stimulation by providing or stimulating different sensory signals, being adapted to the development stage, and being highly interactive to capture attention (Dumoulin et al., 2019; Toledo Del Castillo et al., 2019). Therefore, VR is an innovative distraction technique that should be more effective than traditional methods because it provides visual, auditory, and cognitive stimulation all at once (Wong et al., 2019).

VR is a recent technology that has long been unaffordable and limited by software development and accessibility, preventing its common use in scientific and clinical research (Gold and Mahrer, 2018). Fortunately, the new generation of head-mounted VR equipment is affordable and easy to use, making it a popular and innovative approach for a wide variety of individuals. Furthermore, VR effectively reduces pain caused by punctures in pediatric oncology patients (Nilsson et al., 2009; Birnie et al., 2018; Dunn et al., 2019; Gerceker et al., 2021; Semerci et al., 2021). A recent meta-analysis attempted to analyze seven VR studies but could not demonstrate an advantage of VR because the studies were too heterogeneous to be pooled (Czech et al., 2021).

Therefore, this meta-analysis aimed to examine the effects of VR on pain, fear, and anxiety related to needle procedures in children and adolescents. The results could support the use of VR in such patients.

## Methods

### Literature Search

This study was reported according to the Preferred Reporting Items for Systematic Reviews and Meta-Analyses (PRISMA) guideline (Selcuk, 2019). The PICOS principle was used to build the search strategy (Aslam and Emmanuel, 2010). PubMed, Embase, and the Cochrane Library were searched for potentially eligible studies published up to June 2021 using the MeSH terms of “virtual reality,” “venipuncture,” “neonates,” “pediatric,” “adolescents,” “child,” and other relevant key words, followed by screening based on the inclusion and exclusion criteria. The reference lists of the included studies were screened for additional potentially eligible studies. The search was conducted independently by two investigators. Discrepancies were solved by discussion until a consensus was reached. If the same study population was reported by more than one paper, only the most recent one was selected.

### Eligibility Criteria

The inclusion criteria were (1) patients: ≤ 21 years of age undergoing needle-related procedures, (2) interventions: VR [defined as a fully immersive 3-dimensional computer-generated display in surround stereoscopic vision on a head-mounted display (HMD)]; studies that used 360° videos that were not computer-generated but displayed on a VR HMD were considered eligible as well, (3) outcome: a mean or median score for pain fear, and/or anxiety during the procedure, (4) study type: randomized controlled trial (RCT), including crossover studies, (5) full text available in English, and (6) the full text was accessible. The exclusion criteria were (1) application of VR in non-somatic patient samples, (2) use of audiovisual glasses that offer visual and audio stimulation but do not allow interaction between the user and the computer-generated world, (3) no distinction made between pediatric and adult patients, or (4) study types such as animal studies, reviews, meta-analyses, case reports, case series, dissertations, conference papers, and abstracts.

### Data Extraction

The data were extracted by two investigators according to a pre-specified protocol. Discrepancies in the assessment were solved by discussion until a consensus was reached. If needed, a third investigator was invited to the discussion. Study characteristics (authors, year of publication, country where the study performed, study design, and sample size), patients' characteristics (age and sex), outcome [pain assessed by the Wong-Baker Faces Pain Scale (WBS) (Garra et al., 2010; Drendel et al., 2011), Visual Analog Scale (VAS) (Haefeli and Elfering, 2006), or Faces Pain Scale-Revised (FPS-R) (Hicks et al., 2001), fear assessed by Children's Fear Scale (CFS) (McMurtry et al., 2011), and anxiety assessed by Children's Anxiety Meter (CAM) (Ersig et al., 2013), VAS, or CFS] were extracted.

### Quality of the Evidence

The quality of the included reports was assessed independently by two authors according to Version 2 of the Cochrane risk-of-bias assessment tool for evaluating RCTs (Sterne et al., 2019). Discrepancies in the assessment were resolved through discussion until a consensus was reached.

### Data Synthesis

We used the patients as the primary data source within each study because pain, anxiety, and fear are subjective experiences. Observations of pain and anxiety made by parents, caregivers, and professionals (e.g., nurses, physicians, or investigators) were also entered in the worksheet.

### Statistical Analysis

All analyses were performed using STATA SE 14.0 (StataCorp, College Station, TX, USA). Effects (weighted mean difference [WMD]) and corresponding 95% confidence intervals (CIs) were used to compare the outcomes. Statistical heterogeneity among studies was calculated using Cochran's Q test and the I^2^ index (Higgins et al., 2020). An I^2^ >50% and Q-test P < 0.10 indicated high heterogeneity. This meta-analysis was performed using a random-effects model to avoid overestimation (Higgins et al., 2020). *P*-values < 0.05 were considered statistically different. Results' robustness was tested using sensitivity analyses by sequentially excluding each study and examining whether the WMD and 95%CI remained within the WMD and 95%CI of the overall analysis. Publication bias could not be examined because the number of studies included in each analysis was <10, which could yield misleading results (Higgins et al., 2020).

## Results

### Selection of the Studies

**Figure 1:**
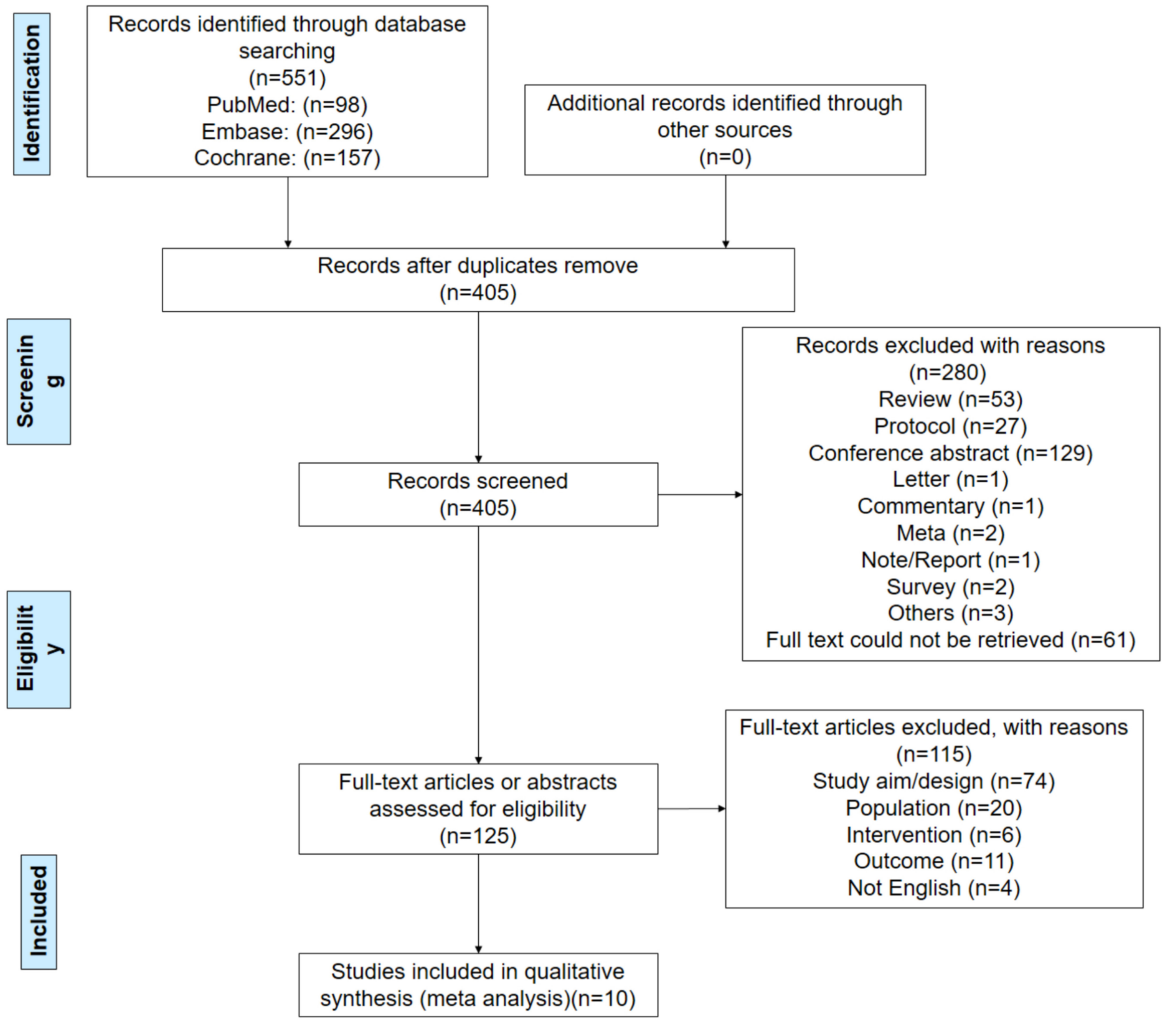
PRISMA 2009 flow diagram.

Figure 1 presents the study selection process. The initial search yielded 551 records, and 146 duplicates were removed. Then, 405 records were screened, and 280 were excluded. Eventually, 125 full-text articles or abstracts were assessed for eligibility, and 115 were excluded (study aim/design, *n* = 74; population, *n* = 20; intervention, *n* = 6; outcomes, *n* = 11; non-English full text, *n* = 4). Finally, 10 RCTs were included. Two studies had two different datasets that could be analyzed (Chan et al., 2019; Ozalp Gerceker et al., 2020).

### Characteristics of the Included Studies

Table 1 presents the characteristics of the studies. The 10 studies included 571 children in the VR group and 575 in the control group. Six studies (seven datasets) were from Turkey (Aydin and Ozyazicioglu, 2019; Inangil et al., 2020; Koc Ozkan and Polat, 2020; Ozalp Gerceker et al., 2020; Gerceker et al., 2021; Semerci et al., 2021), one (two datasets) from Australia (Chan et al., 2019), one from Taiwan (Chen et al., 2020), and two from the United States of America (Gold et al., 2006; Gold and Mahrer, 2018). All participants were children or adolescents. The proportion of males varied from 32.4% to 61.9%.

**Table 1:** Literature search and characteristics of the included studies.

**References**	**Design**	**Country**	**Outcome measurements**	**Population**	**Sample size**		**Age, years**		**Sex, % (male)**	
					**VR**	**SOC**	**VR**	**SOC**	**VR**	**SOC**
Aydin and Ozyazicioglu (2019)	RCT	Turkey	VAS; WBS	Venipuncture procedure	60	60	10.5 (1.14)	10.3 (1.12)	50	51.7
Chan et al. (2019) (Emergency department)	RCT	Australia	FPS-R; VAT	Venipuncture or IV cannulation	64	59	7.9 (6.4-9.9)	8.2 (5.8-10.6)	55	54
Chan et al. (2019) (Pathology)	RCT	Australia	FPS-R; VAT	Venipuncture or IV cannulation	63	66	8.2 (6.3-10.3)	7.4 (5.8-9.1)	60	55
Chen et al. (2020)	RCT	Taiwan	WBS; CFS	IV injections	68	68	9.3 (1.7)	9.0 (1.7)	55.9	57.4
Gerceker et al. (2021)	RCT	Turkey	WBS; CFS; CAM-S	Venous port access	21	21	11.2 (3.1)	11.7 (3.2)	61.9	61.9
Gold et al. (2006)	RCT	USA	VAS; WBS; FPS-R; Childhood anxiety sensitivity index	IV placement	10	10	10.4 (1.58)	10 (1.33)	60	60
Gold and Mahrer (2018)	RCT	USA	VAS; CAS; FPS-Revised; FAS	Blood draws	70	73	15.79 (3)	15.06 (3.23)	52.86	47.95
Inangil et al. (2020)	RCT	Turkey	WBS; CFS	Venipuncture	40	40	9.3 (1.8)	9 (1.7)	50	60
Koc Ozkan and Polat (2020)	RCT	Turkey	CFS; WBS; VAS	Venipuncture procedure	50	50	9.17 (0.97)	9.25 (0.84)	35.1	32.4
Ozalp Gerceker et al. (2020) (Rollercoaster)	RCT	Turkey	WBS; CFS; CAM	Blood draws	45	46	/	/	51.5	56.5
Ozalp Gerceker et al. (2020) (Ocean Rift)	RCT	Turkey	WBS; CFS; CAM	Blood draws	45	46	/	/	53.3	56.5
Semerci et al. (2021)	RCT	Turkey	WBS	Venous port access	35	36	11.69 (3.36)	11.67 (3.55)	54.3	47.2

*VAS, Visual Analog Scale; CAS, Colored Analog Scale; FPS, Faces Pain Scale; FAS, Facial Affective Scale; WBS, Wong-Baker Faces Pain; CFS, Child Fear Scale; CAM, Children's Anxiety Meter; VAT, Visual Analog Thermometer; SOC, Standard of care*.

Supplementary Table 1 presents the quality evaluation of the RCTs. Two studies had a low risk of bias for all items of the ROB2 tool (Chan et al., 2019; Chen et al., 2020), while the other studies had an unclear risk of bias for at least one item (Gold et al., 2006; Gold and Mahrer, 2018; Aydin and Ozyazicioglu, 2019; Inangil et al., 2020; Koc Ozkan and Polat, 2020; Ozalp Gerceker et al., 2020; Gerceker et al., 2021; Semerci et al., 2021).

### Pain

**Figure 2:**
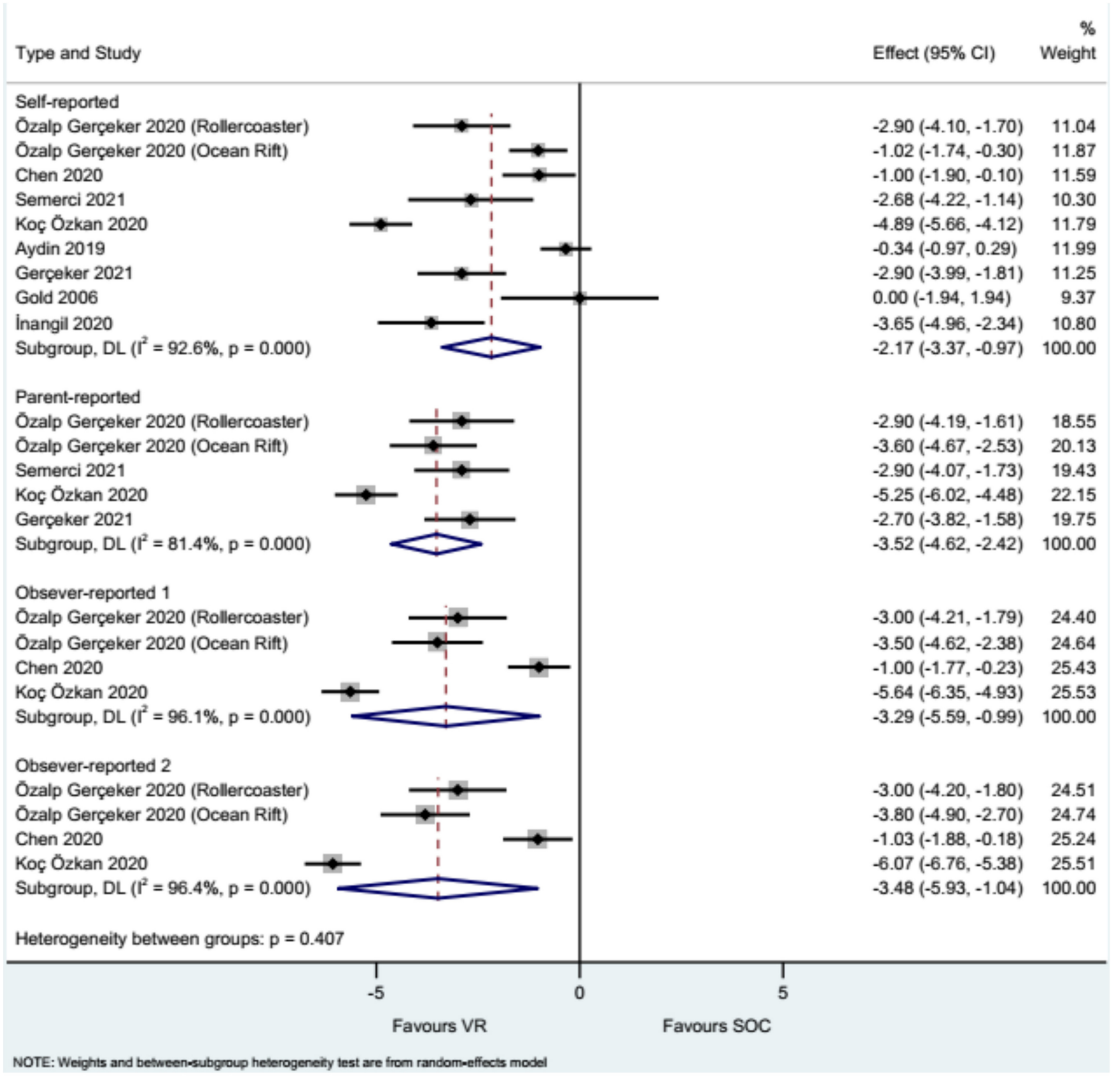
Forest plot of pain assessed by Wong-Baker Faces Pain Scale (WBS). Observer 1: nurses. Observer 2: physicians and investigators.

**Figure 3:**
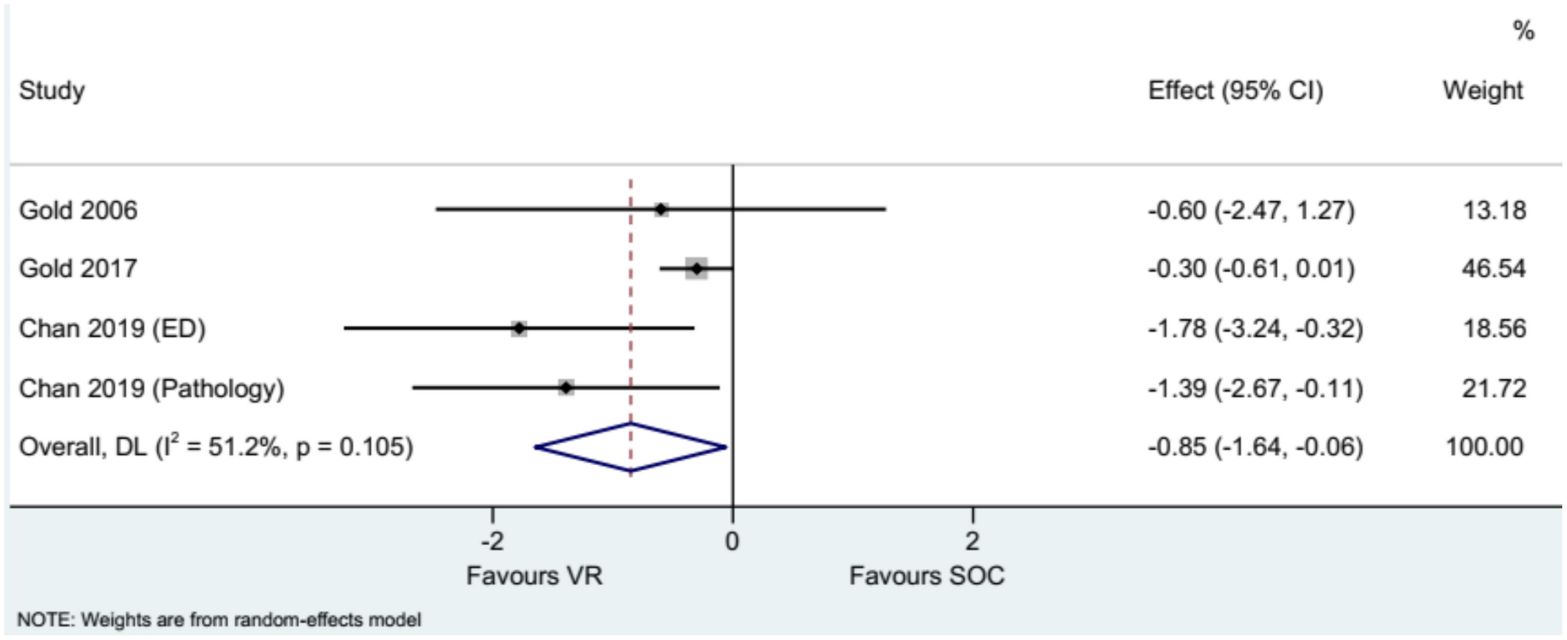
Forest plot of pain assessed by the Faces Pain Scale-Revised (FPS-R).

The studies that used the WBS showed that the use of VR reduced needle-related pain compared with the control intervention when considering self-reported pain (WMD = −2.17, 95%CI: −3.37, −0.97), parent-reported pain (WMD = −3.52, 95%CI: −4.62, −2.42), nurse-reported pain (WMD = −3.29, 95%CI: −5.59, −0.99), and physician/investigator-reported pain (WMD = −3.48, 95%CI: −5.93, −1.04), with heterogeneity observed in all analyses (Figure 2). The summary results of pain assessed using the FPS-R showed that VR reduced needle-related pain compared with controls (WMD = −0.85, 95%CI: −1.64, −0.06), without heterogeneity (Figure 3).

### Fear

**Figure 4:**
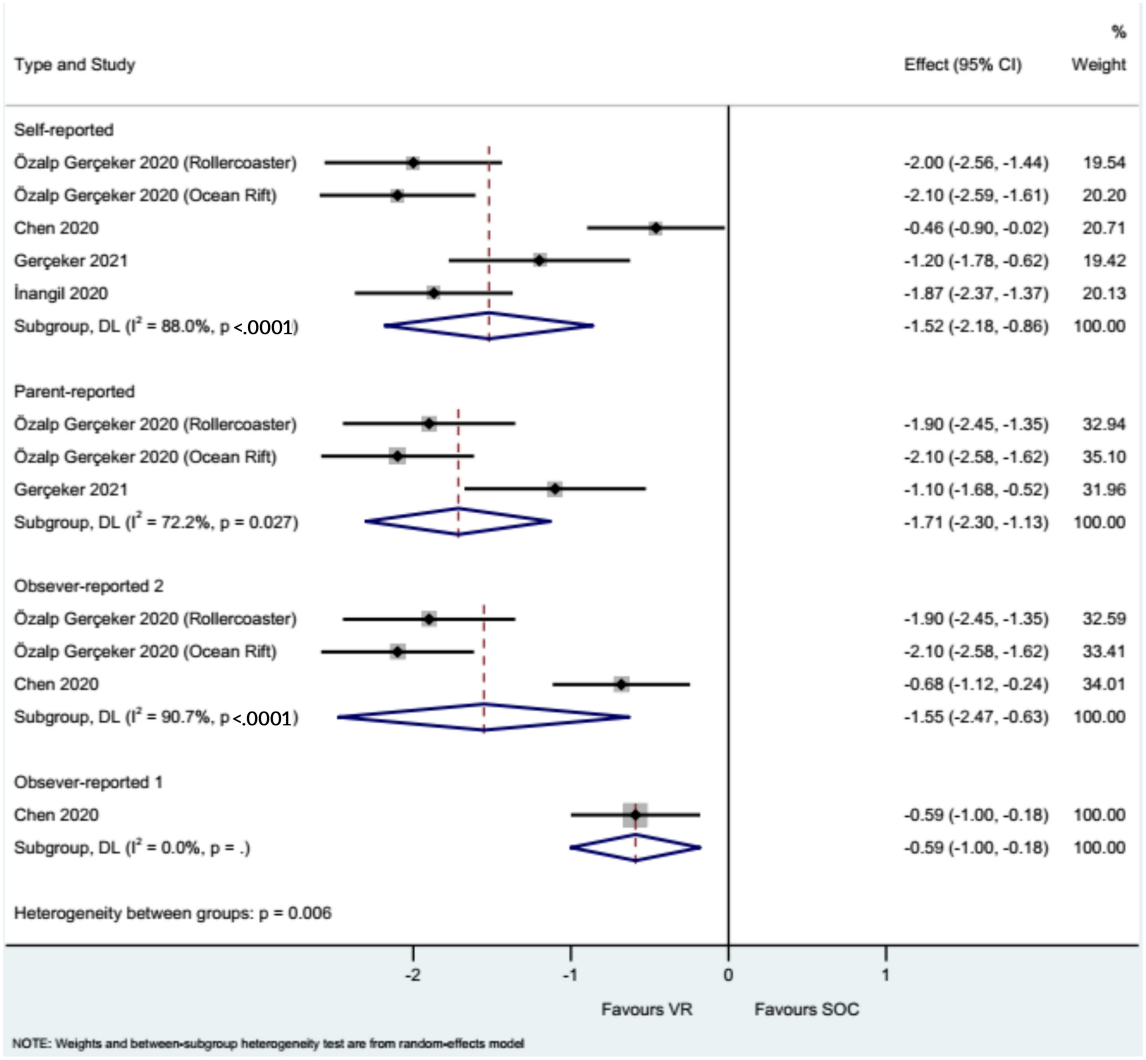
Forest plot of fear assessed by the Child Fear Scale (CFS). Observer 1: nurses. Observer 2: physicians and investigators.

The studies that used the CFS showed that the use of VR reduced needle-related fear compared with the control intervention, either when reported by the children/adolescents (WMD = −1.52, 95%CI: −2.18, −0.86), their parents (WMD = −1.71, 95%CI: −2.30, −1.13), the nurses (WMD = −1.55, 95%CI: −2.47, −0.63), and the physicians or investigators (WMD = −0.59, 95%CI: −1.00, −0.18), with heterogeneity observed in all analyses (Figure 4).

### Anxiety

**Figure 5:**
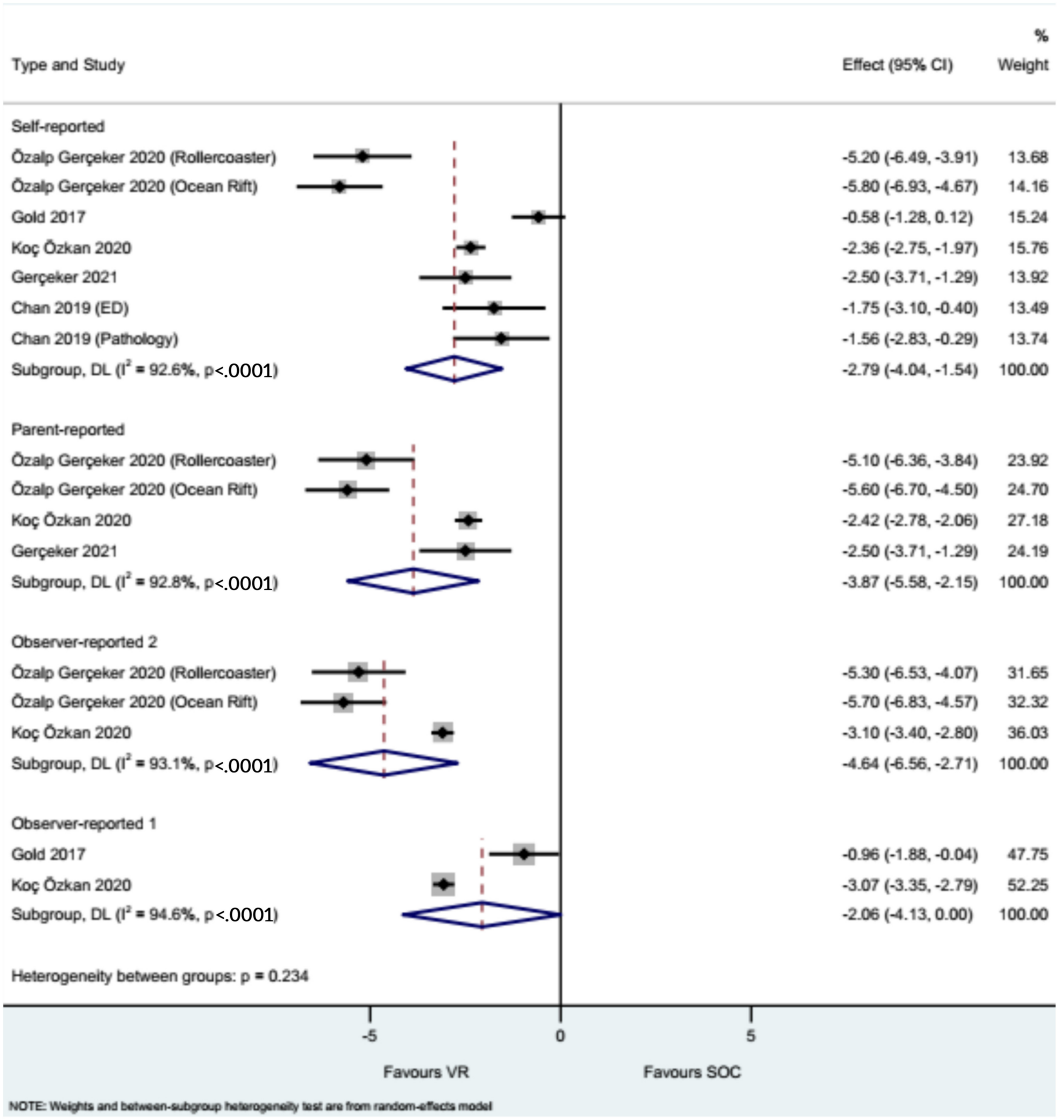
Forest plot of anxiety. Observer 1: nurses. Observer 2: physicians and investigators.

The studies that evaluated anxiety showed that the use of VR reduced needle-related anxiety compared with the control intervention when considering self-reported anxiety (WMD = −2.79, 95%CI: −4.07, −1.54), parent-reported anxiety (WMD = −3.87, 95%CI: −5.58, −2.15), nurse-reported anxiety (WMD = −4.64, 95%CI: −6.56, −2.71), and physician/investigator-reported anxiety (WMD = −2.06, 95%CI: −4.13, −0.00), with heterogeneity observed in all analyses (Figure 5).

### Sensitivity Analyses

Supplementary Figures 1–3 show that all analyses were robust, with the WMDs still within the overall 95%CI when sequentially excluding each study.

## Discussion

Needle-related pain, fear, and anxiety can be a deterrent to treatments in children and adolescents, and sometimes in adults too. VR can be used to manage the poor experience of needle procedures by distracting the patient from the puncture (Dahlquist et al., 2009; Hartling et al., 2013; Gerceker et al., 2018; Inan and Inal, 2019; Koc Ozkan and Polat, 2020). No previous meta-analysis examined VR for needle-related issues. Therefore, this meta-analysis aimed to examine the effects of VR on pain, fear, and anxiety related to needle procedures in children and adolescents. The results suggest that a VR-based intervention could reduce needle-related pain, fear, and anxiety in children and adolescents. The results could support the use of VR in such patients.

Distraction can help manage needle-related pain, fear, and anxiety (Dahlquist et al., 2009; Hartling et al., 2013; Gerceker et al., 2018; Inan and Inal, 2019; Koc Ozkan and Polat, 2020). Previous studies examined traditional methods like music, kaleidoscopes, distraction cards, buzzers, and video games (Dahlquist et al., 2009; Hartling et al., 2013; Gerceker et al., 2018; Inan and Inal, 2019; Koc Ozkan and Polat, 2020). Although these interventions have certain effectiveness, they often stimulate a single sensory pathway. On the other hand, VR is an immersive intervention with sound, moving images, and interactive components that require some thinking and cognitive power (Nilsson et al., 2009; Birnie et al., 2018; Dunn et al., 2019; Gerceker et al., 2021; Semerci et al., 2021). Furthermore, the HMD changes the perception of the surrounding environment. A meta-analysis showed that VR is a useful distraction method in pediatrics (Eijlers et al., 2019) and dental procedures (Lopez-Valverde et al., 2020). Klassen et al. (2008) and Uman et al. (2013) indicated that distraction techniques, including VR, should be used in any needle-related procedures in children. It has been suggested that VR reduces pain perception by reducing the activity of pain-related brain areas (Hughes et al., 2019). Hoffman et al. (2004) also revealed different brain activation patterns by a thermal stimulus in subjects using VR vs. not using VR. Buhle et al. (2012) also showed differences between the effect of VR and the placebo effect. An important result of the present meta-analysis is that VR can reduce anxiety. Anxiety is well-known to intensify pain perception (Woo, 2010). It is supported by a meta-analysis in children (Eijlers et al., 2019) and by another meta-analysis in all ages (Kenney and Milling, 2016). Still, VR could be particularly effective in children because they are often more engaged in magical thinking and imaginative play (Lillard, 1993; Bolton et al., 2002).

In the present study, nearly all analyses showed high heterogeneity, which might be due to the period (2006 to 2021) and the improvements in computers and VR technology over this period. The exact content of the VR could also influence the results since the definition of a pleasurable activity might change among cultures or between children and adolescents (Bertrand et al., 2018; McMichael et al., 2020). The studies also included various populations of patients. Indeed, patients seen once at the emergency department or repeatedly in an oncological setting might have different characteristics of needle-related issues or background anxiety. Czech et al. (2021) observed a similar heterogeneity issue that even prevented them from reaching any conclusion about VR in needle-related procedures. The exact VR content that might be considered as a distractive VR feature can also be different among cultures. In addition, the procedural burden can influence the effectiveness of VR since differences could be observed between children having to undergo a single puncture vs. children (e.g., oncology patients) having to undergo repeated punctures. Therefore, differences in country of origin, ethnicity, VR content, age of the participants, nature of the participants, and underlying patient conditions will influence the results of the included studies and this meta-analysis. Nevertheless, it highlights the need for VR to be highly tailored to the target patient population.

This meta-analysis has strengths. It included many participants who underwent similar interventions and were evaluated using the same tools. Still, this study has limitations. As for all meta-analyses, pooling results will inevitably lead to the inheritance of the limitations of all included studies, and caution must be applied while extrapolating the results. Although the random-effects model was used to minimize the impact of heterogeneity, it cannot be abolished. All the included RCTs included pain levels, either assessed by the children, parents, nurses, and physicians/investigators, but the assessments were non-blinded. Moreover, the interventions themselves were not blinded, which might have affected the performance of the interventions or the needle procedure. Patients' compliance could also affect the results since some studies reported that they had taken off their HMD because they felt distressed. Some studies reported that some patients who met the inclusion criteria refused to participate because of time constraints, suggesting selection bias, as those who chose to participate might have different characteristics compared with those who declined. Finally, the meta-analysis was not registered, and its protocol was not reviewed, which could introduce bias.

In conclusion, this meta-analysis suggests that a VR-based intervention could reduce needle-related pain, fear, and anxiety in children and adolescents. Since the VR technology is ever more affordable and accessible, it could be used in children or adolescents to improve their experience of needle-related procedures or prevent needle phobia, treatment avoidance, and poor adherence.

## Data Availability Statement

The original contributions presented in the study are included in the article/Supplementary Material, further inquiries can be directed to the corresponding author.

## Author Contributions

All authors listed have made a substantial, direct, and intellectual contribution to the work and approved it for publication.

## Conflict of Interest

The authors declare that the research was conducted in the absence of any commercial or financial relationships that could be construed as a potential conflict of interest.

## Publisher's Note

All claims expressed in this article are solely those of the authors and do not necessarily represent those of their affiliated organizations, or those of the publisher, the editors and the reviewers. Any product that may be evaluated in this article, or claim that may be made by its manufacturer, is not guaranteed or endorsed by the publisher.

## Supplementary Material

Supplementary Figure 1 Sensitivity analysis of pain assessed by the Wong-Baker Faces Pain Scale (WBS). **(A)** Self-report. **(B)** Parent-report. **(C)** Observer 1-report. **(D)** Observer 2-report. Observer 1: nurses. Observer 2: physicians and investigators.

Supplementary Figure 2 Sensitivity analysis of fear assessed by Child Fear Scale (CFS). **(A)** Self-report. **(B)** Parent-report. **(C)** Observer 2-report (physicians/investigators).

Supplementary Figure 3 Sensitivity analysis of anxiety. **(A)** Self-report. **(B)** Parent-report. **(C)** Observer 1-report **(D)** Observer 2-report. Observer 1: nurses. Observer 2: physicians and investigators.
