# Supplementary figures and images for: Effects of Virtual Reality-Based Distraction of Pain, Fear, and Anxiety During Needle-Related Procedures in Children and Adolescents
Source: Front Psychol. 2022 Apr 19;13:842847. doi: 10.3389/fpsyg.2022.842847 (PMC9063726; doi:10.3389/fpsyg.2022.842847)

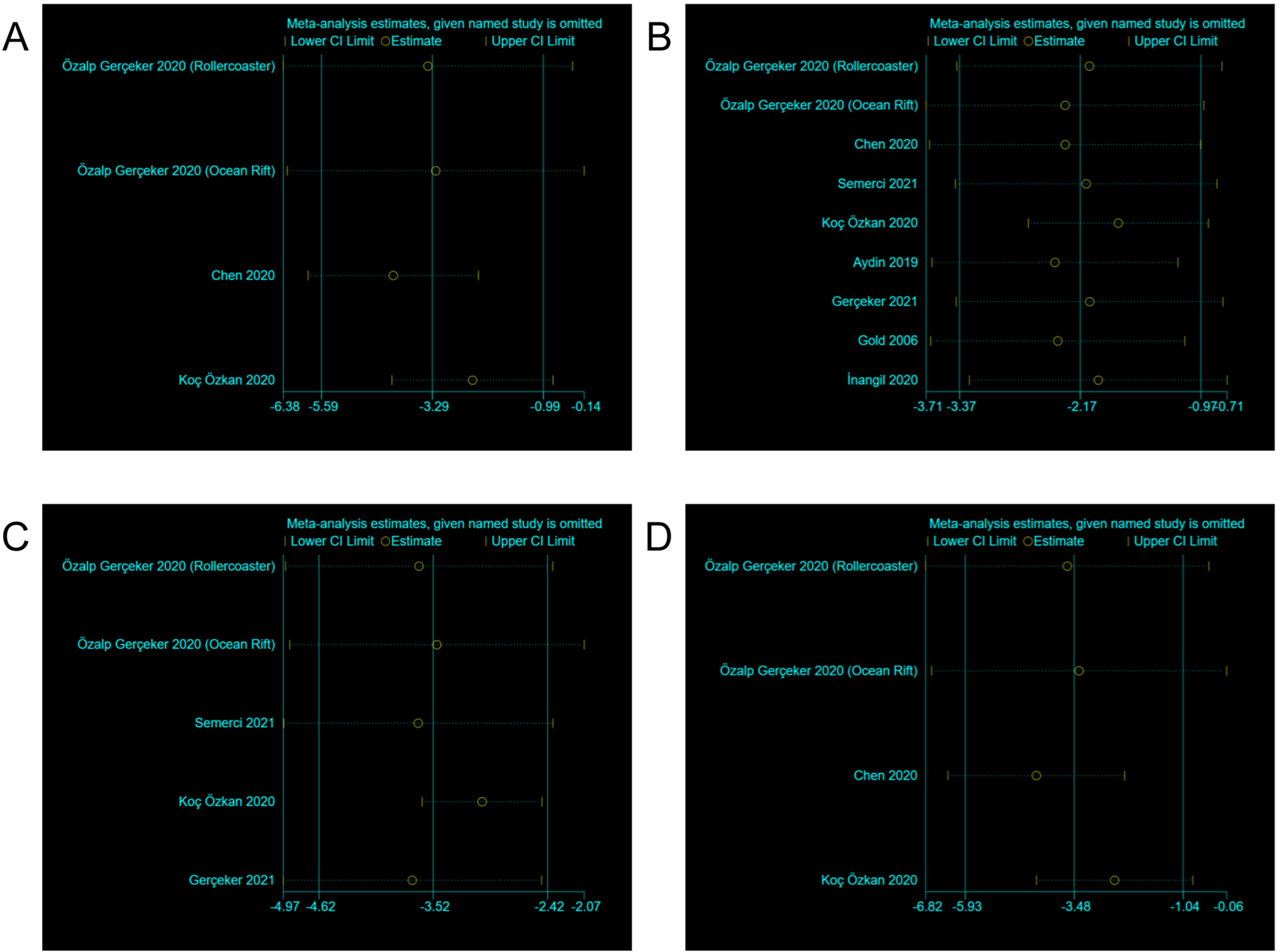

Supplement: Supplementary Figure 1 — Sensitivity analysis of pain assessed by the Wong-Baker Faces Pain Scale (WBS). (A) Self-report. (B) Parent-report. (C) Observer 1-report. (D) Observer 2-report. Observer 1: nurses. Observer 2: physicians and investigators. [file Image_1.jpg]

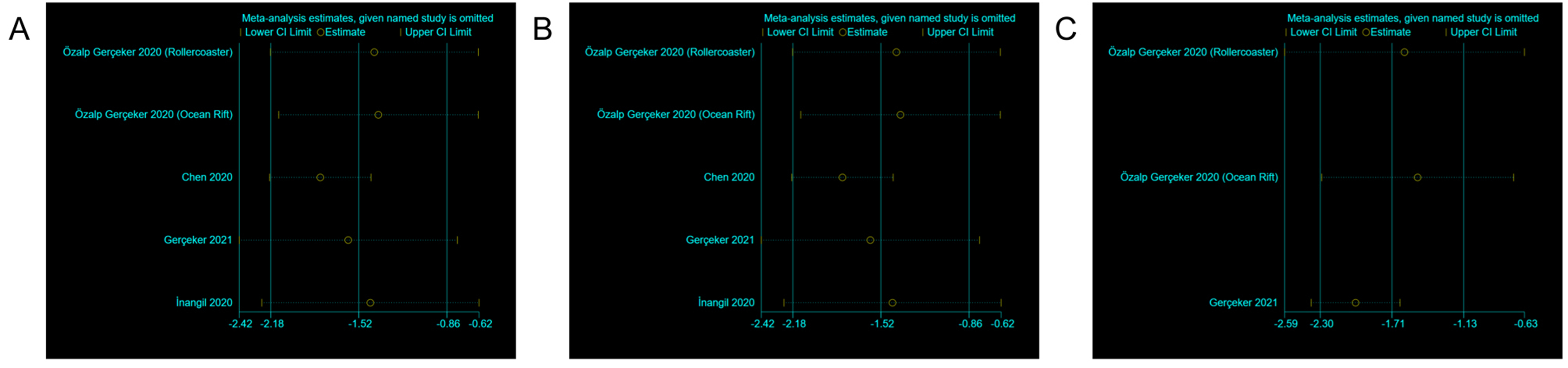

Supplement: Supplementary Figure 2 — Sensitivity analysis of fear assessed by Child Fear Scale (CFS). (A) Self-report. (B) Parent-report. (C) Observer 2-report (physicians/investigators). [file Image_2.jpg]

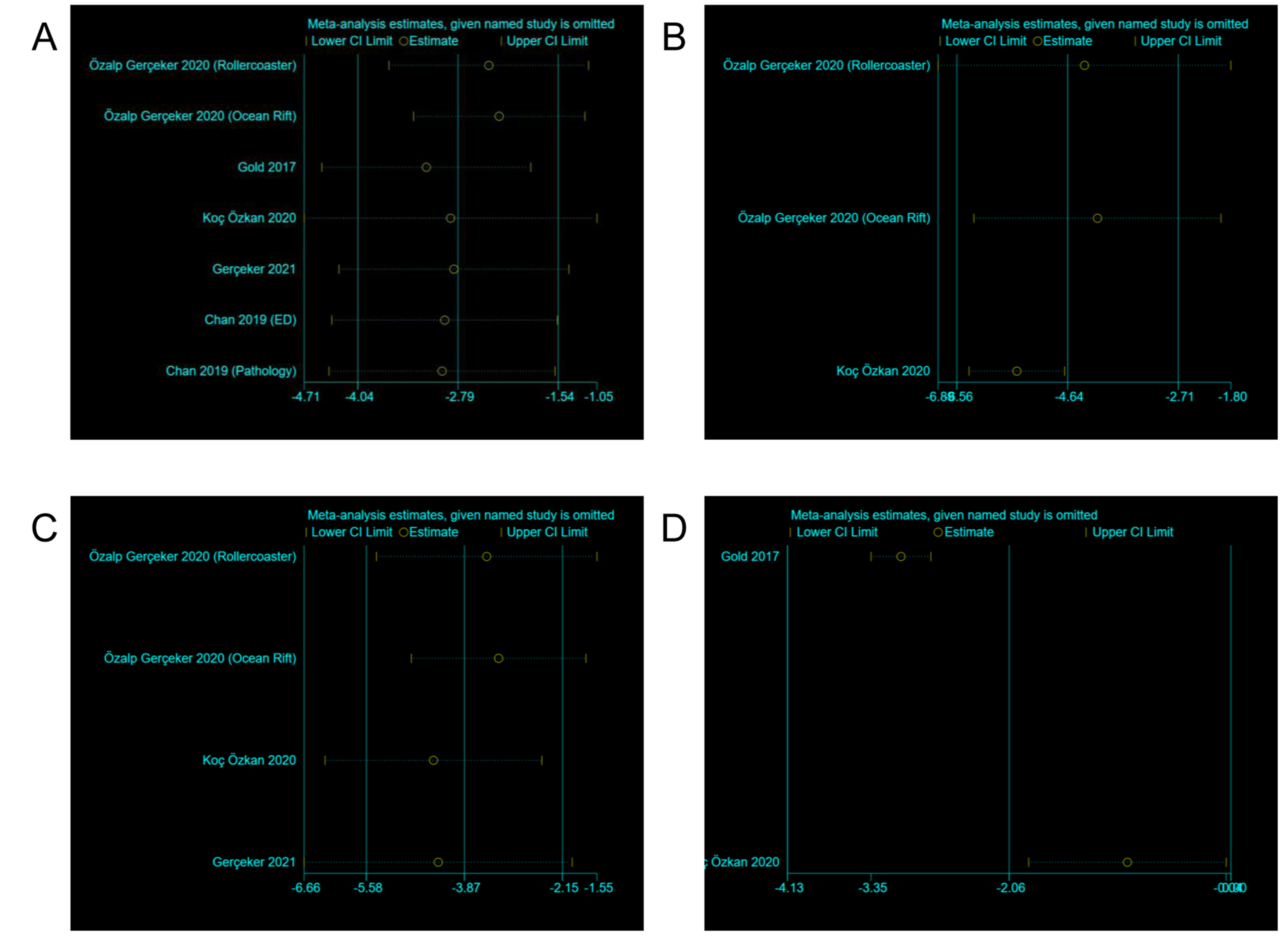

Supplement: Supplementary Figure 3 — Sensitivity analysis of anxiety. (A) Self-report. (B) Parent-report. (C) Observer 1-report (D) Observer 2-report. Observer 1: nurses. Observer 2: physicians and investigators. [file Image_3.jpg]
