# Supplementary material for: Effects of Virtual Reality-Based Distraction of Pain, Fear, and Anxiety During Needle-Related Procedures in Children and Adolescents
Source: Front Psychol. 2022 Apr 19;13:842847. doi: 10.3389/fpsyg.2022.842847 (PMC9063726; doi:10.3389/fpsyg.2022.842847)
Supplement: Supplementary file 5 [file Data_Sheet_2.pdf]

Supplementary Table S1. ROB 2.0 criteria for RCTs

Study	Bias arising from the randomization process	Bias due to deviations from intended interventions	Bias due to missing outcome data	Bias in measurement of the outcome	Bias in selection of the reported result	Overall bias
Aydin 2019 (33)	Unclear	Low	Low	Low	Low	Low
Chan 2019 (8)	Low	Low	Low	Low	Low	Low
Chen 2020 (35)	Low	Low	Low	Low	Low	Low
Gerçeker 2021 (19)	Unclear	Unclear	Low	Low	Low	Unclear
Gold 2006 (36)	Unclear	Unclear	Low	Low	Low	Unclear
Gold 2018 (17)	Unclear	Low	Low	Low	Low	Low
İnangil 2020 (34)	Low	Unclear	Low	Low	Low	Low
Koç Özkan 2020 (7)	Low	Unclear	Low	Low	Low	Low
Özalp Gerçeker 2020 (9)	Low	Unclear	Low	Low	Low	Low
Semerci 2021 (21)	Low	Unclear	Low	Low	Low	Low
